# Bis{benzyl 3-[(1*H*-indol-3-yl)methyl­idene]dithio­carbazato-κ^2^ *N* ^3^,*S*}palladium(II) *N*,*N*-dimethyl­formamide disolvate

**DOI:** 10.1107/S1600536810051780

**Published:** 2010-12-15

**Authors:** Hamid Khaledi, Hapipah Mohd Ali

**Affiliations:** a Department of Chemistry, University of Malaya, 50603 Kuala Lumpur, Malaysia

## Abstract

In the title compound, [Pd(C_17_H_14_N_3_S_2_)_2_]·2C_3_H_7_NO, the deprotonated Schiff base ligand acts as an *N*,*S*-bidentate chelate, forming a five-membered ring with the metal atom. The Pd^II^ ion, located on an inversion center, is four-coordinated by two of the Schiff base ligands in a square-planar geometry. In the crystal, the indolic NH groups are bonded to the dimethyl­formamide (DMF) solvent mol­ecules *via* an N—H⋯O inter­action. In addition, C—H⋯S inter­actions are observed.

## Related literature

For the crystal structure of the ligand, see: Khaledi *et al.* (2008 ▶). For the isotypic Cu(II) analog, see: Khaledi *et al.* (2009 ▶). For the Pd^II^ complex of the acetone Schiff base of *S*-methyl­dithio­carbazate, see: Ali *et al.* (2002 ▶).
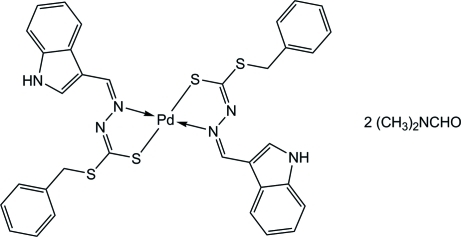


## Experimental

### 

#### Crystal data

[Pd(C_17_H_14_N_3_S_2_)_2_]·2C_3_H_7_NO
*M* *_r_* = 901.46
Monoclinic, 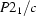

*a* = 10.509 (4) Å
*b* = 20.320 (7) Å
*c* = 10.925 (4) Å
β = 117.577 (5)°
*V* = 2067.8 (12) Å^3^
*Z* = 2
Mo *K*α radiation
μ = 0.70 mm^−1^
*T* = 296 K
0.30 × 0.15 × 0.03 mm

#### Data collection

Bruker APEXII CCD diffractometer
Absorption correction: multi-scan (*SADABS*; Sheldrick, 1996 ▶) *T*_min_ = 0.818, *T*_max_ = 0.979
11353 measured reflections
4486 independent reflections
3411 reflections with *I* > 2σ(*I*)
*R*_int_ = 0.024

#### Refinement

*R*[*F* ^2^ > 2σ(*F* ^2^)] = 0.030
*wR*(*F* ^2^) = 0.074
*S* = 1.02
4486 reflections
255 parameters
1 restraint
H atoms treated by a mixture of independent and constrained refinement
Δρ_max_ = 0.27 e Å^−3^
Δρ_min_ = −0.31 e Å^−3^

### 

Data collection: *APEX2* (Bruker, 2007 ▶); cell refinement: *SAINT* (Bruker, 2007 ▶); data reduction: *SAINT*; program(s) used to solve structure: *SHELXS97* (Sheldrick, 2008 ▶); program(s) used to refine structure: *SHELXL97* (Sheldrick, 2008 ▶); molecular graphics: *X-SEED* (Barbour, 2001 ▶); software used to prepare material for publication: *SHELXL97* and *publCIF* (Westrip, 2010 ▶).

## Supplementary Material

Crystal structure: contains datablocks I, global. DOI: 10.1107/S1600536810051780/pv2368sup1.cif

Structure factors: contains datablocks I. DOI: 10.1107/S1600536810051780/pv2368Isup2.hkl

Additional supplementary materials:  crystallographic information; 3D view; checkCIF report

## Figures and Tables

**Table 1:** Hydrogen-bond geometry (Å, °)

*D*—H⋯*A*	*D*—H	H⋯*A*	*D*⋯*A*	*D*—H⋯*A*
N1—H1*N*⋯O1	0.84 (2)	1.91 (2)	2.749 (3)	174 (3)
C9—H9⋯S1^i^	0.93	2.60	3.279 (2)	130

Symmetry code: (i) 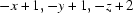.

**Fig. 1.**
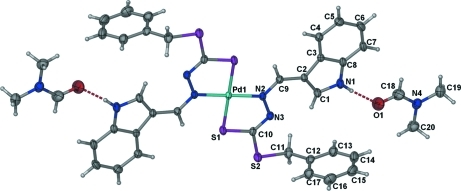
Thermal ellipsoid plot of the title compound at the 30% probability level. Hydrogen atoms are drawn as spheres of arbitrary radius.

## supplementary crystallographic information

### Comment

The title compound is isostructural with the Cu^II^ complex of the Schiff base ligand (Khaledi *et al.*, 2009). The palladium(II) ion is four-coordinated by two azomethine nitrogen and two thioamide sulfur atoms in a *trans*-square planar geometry. It has been suggested that the square planar geometry of the Schiff bases of *S*-alkyldithiocarbazate is *trans* when they are derived from aldehydes, whereas the ketone derivatives show *cis* geometry (Ali *et al.*, 2002). Similar to the analogous Cu^II^ complex, the indole amino groups in the present structure are hydrogen bonded to the co-crystallized DMF molecules. Moreover, non-classical hydrogen bonds, C—H···N, C—H···O and C—H···S, are observed in the structure.

### Experimental

The Schiff base ligand was prepared as reported previously (Khaledi *et al*., 2008). A solution of palladium(II) acetate (0.224 g, 1 mmol) in ethanol (30 ml) was added to an ethanolic solution (30 ml) of the ligand (0.65 g, 2 mmol) containing a few drops of triethylamine. The mixture was refluxed for an hour, then cooled to room temperature. The resulting brown solid was filtered, washed with cold ethanol and dried over siliga-gel. The title crystals were obtained by slow evaporation of a solution of the solid in DMF.

### Refinement

The C-bound H atoms were placed at calculated positions (C–H 0.93–0.97 Å) and were treated as riding on their parent C atoms. The N-bound H atom was located in a difference Fourier map, and was refined with a distance restraint of N–H 0.86±0.02. For all H atoms, *U*_iso_(H) was set to 1.2–1.5 *U*_eq_(carrier atom).

### Crystal data

**Table:**

[Pd(C_17_H_14_N_3_S_2_)_2_]·2C_3_H_7_NO	*F*(000) = 928
*M**_r_* = 901.46	*D*_x_ = 1.448 Mg m^−^^3^
Monoclinic, *P*2_1_/*c*	Mo *K*α radiation, λ = 0.71073 Å
Hall symbol: -P 2ybc	Cell parameters from 3977 reflections
*a* = 10.509 (4) Å	θ = 2.3–29.4°
*b* = 20.320 (7) Å	µ = 0.70 mm^−^^1^
*c* = 10.925 (4) Å	*T* = 296 K
β = 117.577 (5)°	Plate, red
*V* = 2067.8 (12) Å^3^	0.30 × 0.15 × 0.03 mm
*Z* = 2	

### Data collection

**Table:**

Bruker APEXII CCD diffractometer	4486 independent reflections
Radiation source: fine-focus sealed tube	3411 reflections with *I* > 2σ(*I*)
graphite	*R*_int_ = 0.024
φ and ω scans	θ_max_ = 27.0°, θ_min_ = 2.0°
Absorption correction: multi-scan (*SADABS*; Sheldrick, 1996)	*h* = −13→12
*T*_min_ = 0.818, *T*_max_ = 0.979	*k* = −25→25
11353 measured reflections	*l* = −11→13

### Refinement

**Table:**

Refinement on *F*^2^	Primary atom site location: structure-invariant direct methods
Least-squares matrix: full	Secondary atom site location: difference Fourier map
*R*[*F*^2^ > 2σ(*F*^2^)] = 0.030	Hydrogen site location: inferred from neighbouring sites
*wR*(*F*^2^) = 0.074	H atoms treated by a mixture of independent and constrained refinement
*S* = 1.02	*w* = 1/[σ^2^(*F*_o_^2^) + (0.0332*P*)^2^ + 0.5126*P*] where *P* = (*F*_o_^2^ + 2*F*_c_^2^)/3
4486 reflections	(Δ/σ)_max_ = 0.001
255 parameters	Δρ_max_ = 0.27 e Å^−^^3^
1 restraint	Δρ_min_ = −0.31 e Å^−^^3^

### Special details

**Table:**

Geometry. All e.s.d.'s (except the e.s.d. in the dihedral angle between two l.s. planes) are estimated using the full covariance matrix. The cell e.s.d.'s are taken into account individually in the estimation of e.s.d.'s in distances, angles and torsion angles; correlations between e.s.d.'s in cell parameters are only used when they are defined by crystal symmetry. An approximate (isotropic) treatment of cell e.s.d.'s is used for estimating e.s.d.'s involving l.s. planes.
Refinement. Refinement of *F*^2^ against ALL reflections. The weighted *R*-factor *wR* and goodness of fit *S* are based on *F*^2^, conventional *R*-factors *R* are based on *F*, with *F* set to zero for negative *F*^2^. The threshold expression of *F*^2^ > σ(*F*^2^) is used only for calculating *R*-factors(gt) *etc*. and is not relevant to the choice of reflections for refinement. *R*-factors based on *F*^2^ are statistically about twice as large as those based on *F*, and *R*- factors based on ALL data will be even larger.

### Fractional atomic coordinates and isotropic or equivalent isotropic displacement parameters (Å^2^)

**Table:**

	*x*	*y*	*z*	*U*_iso_*/*U*_eq_	
Pd1	0.5000	0.5000	1.0000	0.03881 (8)	
S1	0.50770 (7)	0.38703 (3)	1.00654 (7)	0.05372 (16)	
S2	0.60819 (8)	0.29517 (3)	0.86690 (7)	0.05937 (18)	
N1	0.7185 (2)	0.50542 (10)	0.5369 (2)	0.0550 (5)	
H1N	0.753 (3)	0.4818 (12)	0.496 (3)	0.066*	
N2	0.56951 (19)	0.48716 (8)	0.85694 (19)	0.0422 (4)	
N3	0.60342 (19)	0.42432 (8)	0.82622 (19)	0.0438 (4)	
C1	0.6788 (3)	0.48235 (11)	0.6292 (3)	0.0517 (6)	
H1	0.6771	0.4381	0.6505	0.062*	
C2	0.6405 (2)	0.53413 (10)	0.6881 (2)	0.0438 (5)	
C3	0.6598 (2)	0.59335 (10)	0.6243 (2)	0.0434 (5)	
C4	0.6429 (3)	0.66054 (11)	0.6388 (3)	0.0547 (6)	
H4	0.6111	0.6755	0.7005	0.066*	
C5	0.6739 (3)	0.70436 (13)	0.5604 (3)	0.0667 (7)	
H5	0.6642	0.7493	0.5703	0.080*	
C6	0.7198 (3)	0.68253 (13)	0.4660 (3)	0.0692 (8)	
H6	0.7388	0.7132	0.4134	0.083*	
C7	0.7374 (3)	0.61730 (13)	0.4495 (3)	0.0595 (6)	
H7	0.7678	0.6029	0.3865	0.071*	
C8	0.7080 (2)	0.57292 (11)	0.5304 (2)	0.0479 (5)	
C9	0.5902 (2)	0.53485 (11)	0.7881 (2)	0.0449 (5)	
H9	0.5681	0.5765	0.8081	0.054*	
C10	0.5759 (2)	0.37785 (10)	0.8902 (2)	0.0424 (5)	
C11	0.6598 (3)	0.29661 (12)	0.7301 (3)	0.0587 (6)	
H11A	0.6411	0.2533	0.6880	0.070*	
H11B	0.5969	0.3273	0.6602	0.070*	
C12	0.8120 (3)	0.31475 (10)	0.7668 (3)	0.0514 (6)	
C13	0.8432 (3)	0.32943 (13)	0.6598 (3)	0.0641 (7)	
H13	0.7699	0.3284	0.5694	0.077*	
C14	0.9801 (3)	0.34555 (15)	0.6844 (4)	0.0792 (9)	
H14	0.9987	0.3548	0.6108	0.095*	
C15	1.0875 (3)	0.34793 (16)	0.8149 (4)	0.0858 (10)	
H15	1.1798	0.3595	0.8316	0.103*	
C16	1.0600 (3)	0.33329 (16)	0.9226 (4)	0.0884 (10)	
H16	1.1340	0.3348	1.0126	0.106*	
C17	0.9222 (3)	0.31618 (14)	0.8987 (3)	0.0706 (7)	
H17	0.9050	0.3057	0.9726	0.085*	
O1	0.8241 (4)	0.43495 (13)	0.3888 (3)	0.1248 (10)	
N4	0.9096 (2)	0.42640 (11)	0.2347 (2)	0.0634 (6)	
C18	0.8601 (4)	0.45922 (17)	0.3077 (4)	0.0946 (11)	
H18	0.8520	0.5046	0.2964	0.113*	
C19	0.9457 (3)	0.45852 (16)	0.1364 (3)	0.0827 (9)	
H19A	0.9369	0.5053	0.1420	0.124*	
H19B	0.8815	0.4438	0.0448	0.124*	
H19C	1.0426	0.4477	0.1574	0.124*	
C20	0.9206 (3)	0.35570 (14)	0.2455 (3)	0.0785 (8)	
H20A	0.9153	0.3418	0.3269	0.118*	
H20B	1.0106	0.3420	0.2511	0.118*	
H20C	0.8432	0.3363	0.1656	0.118*	

### Atomic displacement parameters (Å^2^)

**Table:**

	*U*^11^	*U*^22^	*U*^33^	*U*^12^	*U*^13^	*U*^23^
Pd1	0.04047 (13)	0.04106 (13)	0.04411 (14)	0.00056 (10)	0.02740 (11)	0.00044 (11)
S1	0.0737 (4)	0.0436 (3)	0.0685 (4)	−0.0004 (3)	0.0537 (4)	0.0015 (3)
S2	0.0807 (4)	0.0410 (3)	0.0797 (5)	−0.0021 (3)	0.0569 (4)	−0.0023 (3)
N1	0.0705 (13)	0.0540 (12)	0.0608 (13)	−0.0030 (10)	0.0477 (11)	−0.0044 (10)
N2	0.0469 (10)	0.0428 (10)	0.0467 (10)	0.0017 (7)	0.0301 (9)	−0.0002 (8)
N3	0.0503 (10)	0.0407 (9)	0.0511 (11)	0.0002 (8)	0.0325 (9)	−0.0031 (8)
C1	0.0656 (15)	0.0462 (12)	0.0603 (15)	−0.0024 (11)	0.0434 (13)	0.0003 (11)
C2	0.0495 (12)	0.0444 (12)	0.0480 (13)	0.0014 (10)	0.0314 (11)	0.0026 (10)
C3	0.0426 (11)	0.0470 (12)	0.0466 (12)	0.0015 (10)	0.0257 (10)	0.0046 (10)
C4	0.0573 (14)	0.0507 (13)	0.0664 (16)	0.0053 (11)	0.0374 (13)	0.0056 (12)
C5	0.0681 (17)	0.0505 (14)	0.089 (2)	0.0026 (12)	0.0431 (16)	0.0135 (14)
C6	0.0660 (17)	0.0691 (17)	0.082 (2)	−0.0025 (13)	0.0426 (16)	0.0267 (15)
C7	0.0599 (15)	0.0741 (17)	0.0586 (16)	−0.0024 (13)	0.0394 (13)	0.0115 (13)
C8	0.0475 (12)	0.0546 (13)	0.0490 (13)	−0.0030 (10)	0.0287 (11)	0.0032 (11)
C9	0.0513 (13)	0.0404 (11)	0.0528 (14)	0.0030 (10)	0.0323 (12)	0.0018 (10)
C10	0.0432 (11)	0.0430 (11)	0.0487 (13)	−0.0026 (9)	0.0278 (11)	−0.0040 (10)
C11	0.0702 (16)	0.0537 (14)	0.0667 (16)	−0.0089 (12)	0.0440 (14)	−0.0186 (12)
C12	0.0610 (15)	0.0404 (11)	0.0645 (16)	0.0026 (10)	0.0389 (14)	−0.0099 (11)
C13	0.0680 (17)	0.0654 (16)	0.0694 (17)	0.0005 (13)	0.0409 (15)	−0.0041 (14)
C14	0.076 (2)	0.082 (2)	0.101 (3)	0.0005 (17)	0.059 (2)	0.0081 (19)
C15	0.0625 (19)	0.082 (2)	0.122 (3)	0.0053 (16)	0.050 (2)	0.009 (2)
C16	0.0637 (19)	0.095 (2)	0.089 (2)	0.0093 (17)	0.0210 (18)	−0.0057 (19)
C17	0.0734 (19)	0.0774 (18)	0.0687 (19)	0.0053 (15)	0.0393 (17)	−0.0040 (15)
O1	0.202 (3)	0.120 (2)	0.1058 (19)	0.0498 (19)	0.116 (2)	0.0098 (16)
N4	0.0643 (13)	0.0729 (14)	0.0570 (13)	0.0028 (11)	0.0315 (12)	−0.0111 (11)
C18	0.136 (3)	0.080 (2)	0.086 (2)	0.026 (2)	0.067 (2)	−0.0029 (19)
C19	0.079 (2)	0.095 (2)	0.083 (2)	−0.0068 (17)	0.0451 (19)	−0.0070 (18)
C20	0.090 (2)	0.0727 (19)	0.073 (2)	0.0108 (16)	0.0386 (18)	−0.0092 (16)

### Geometric parameters (Å, °)

**Table:**

Pd1—N2^i^	2.0252 (18)	C9—H9	0.9300
Pd1—N2	2.0252 (18)	C11—C12	1.505 (3)
Pd1—S1	2.2969 (10)	C11—H11A	0.9700
Pd1—S1^i^	2.2969 (10)	C11—H11B	0.9700
S1—C10	1.735 (2)	C12—C17	1.369 (4)
S2—C10	1.755 (2)	C12—C13	1.384 (3)
S2—C11	1.810 (2)	C13—C14	1.376 (4)
N1—C1	1.342 (3)	C13—H13	0.9300
N1—C8	1.375 (3)	C14—C15	1.349 (4)
N1—H1N	0.840 (16)	C14—H14	0.9300
N2—C9	1.305 (3)	C15—C16	1.368 (4)
N2—N3	1.407 (2)	C15—H15	0.9300
N3—C10	1.285 (3)	C16—C17	1.391 (4)
C1—C2	1.387 (3)	C16—H16	0.9300
C1—H1	0.9300	C17—H17	0.9300
C2—C9	1.417 (3)	O1—C18	1.218 (4)
C2—C3	1.451 (3)	N4—C18	1.317 (4)
C3—C4	1.395 (3)	N4—C20	1.442 (3)
C3—C8	1.401 (3)	N4—C19	1.449 (4)
C4—C5	1.375 (3)	C18—H18	0.9300
C4—H4	0.9300	C19—H19A	0.9600
C5—C6	1.398 (4)	C19—H19B	0.9600
C5—H5	0.9300	C19—H19C	0.9600
C6—C7	1.362 (4)	C20—H20A	0.9600
C6—H6	0.9300	C20—H20B	0.9600
C7—C8	1.394 (3)	C20—H20C	0.9600
C7—H7	0.9300		
			
N2^i^—Pd1—N2	179.999 (1)	S1—C10—S2	112.47 (12)
N2^i^—Pd1—S1	97.17 (5)	C12—C11—S2	118.18 (19)
N2—Pd1—S1	82.83 (5)	C12—C11—H11A	107.8
N2^i^—Pd1—S1^i^	82.83 (5)	S2—C11—H11A	107.8
N2—Pd1—S1^i^	97.17 (5)	C12—C11—H11B	107.8
S1—Pd1—S1^i^	180.0	S2—C11—H11B	107.8
C10—S1—Pd1	96.04 (7)	H11A—C11—H11B	107.1
C10—S2—C11	104.82 (11)	C17—C12—C13	118.0 (2)
C1—N1—C8	109.95 (19)	C17—C12—C11	124.2 (2)
C1—N1—H1N	123.9 (19)	C13—C12—C11	117.8 (2)
C8—N1—H1N	125.9 (19)	C14—C13—C12	121.4 (3)
C9—N2—N3	114.08 (17)	C14—C13—H13	119.3
C9—N2—Pd1	124.40 (15)	C12—C13—H13	119.3
N3—N2—Pd1	121.50 (12)	C15—C14—C13	120.2 (3)
C10—N3—N2	113.06 (17)	C15—C14—H14	119.9
N1—C1—C2	110.0 (2)	C13—C14—H14	119.9
N1—C1—H1	125.0	C14—C15—C16	119.6 (3)
C2—C1—H1	125.0	C14—C15—H15	120.2
C1—C2—C9	131.1 (2)	C16—C15—H15	120.2
C1—C2—C3	105.77 (19)	C15—C16—C17	120.6 (3)
C9—C2—C3	123.12 (19)	C15—C16—H16	119.7
C4—C3—C8	118.8 (2)	C17—C16—H16	119.7
C4—C3—C2	134.7 (2)	C12—C17—C16	120.2 (3)
C8—C3—C2	106.49 (18)	C12—C17—H17	119.9
C5—C4—C3	118.9 (2)	C16—C17—H17	119.9
C5—C4—H4	120.5	C18—N4—C20	119.6 (3)
C3—C4—H4	120.5	C18—N4—C19	122.2 (3)
C4—C5—C6	121.1 (2)	C20—N4—C19	118.1 (2)
C4—C5—H5	119.5	O1—C18—N4	125.4 (3)
C6—C5—H5	119.5	O1—C18—H18	117.3
C7—C6—C5	121.4 (2)	N4—C18—H18	117.3
C7—C6—H6	119.3	N4—C19—H19A	109.5
C5—C6—H6	119.3	N4—C19—H19B	109.5
C6—C7—C8	117.5 (2)	H19A—C19—H19B	109.5
C6—C7—H7	121.2	N4—C19—H19C	109.5
C8—C7—H7	121.2	H19A—C19—H19C	109.5
N1—C8—C7	129.9 (2)	H19B—C19—H19C	109.5
N1—C8—C3	107.81 (18)	N4—C20—H20A	109.5
C7—C8—C3	122.3 (2)	N4—C20—H20B	109.5
N2—C9—C2	131.1 (2)	H20A—C20—H20B	109.5
N2—C9—H9	114.4	N4—C20—H20C	109.5
C2—C9—H9	114.4	H20A—C20—H20C	109.5
N3—C10—S1	126.36 (16)	H20B—C20—H20C	109.5
N3—C10—S2	121.17 (16)		

Symmetry codes: (i) −*x*+1, −*y*+1, −*z*+2.

### Hydrogen-bond geometry (Å, °)

**Table:**

*D*—H···*A*	*D*—H	H···*A*	*D*···*A*	*D*—H···*A*
N1—H1N···O1	0.84 (2)	1.91 (2)	2.749 (3)	174 (3)
C1—H1···N3	0.93	2.40	2.869 (3)	111.
C17—H17···S2	0.93	2.79	3.183 (3)	107.
C20—H20A···O1	0.96	2.36	2.747 (3)	104.
C9—H9···S1^i^	0.93	2.60	3.279 (2)	130.

Symmetry codes: (i) −*x*+1, −*y*+1, −*z*+2.
